# Effect of Cold Rolling on Microstructural and Mechanical Properties of a Dual-Phase Steel for Automotive Field

**DOI:** 10.3390/ma15217482

**Published:** 2022-10-25

**Authors:** Emilio Bassini, Giulio Marchese, Antonio Sivo, Pietro Antonio Martelli, Alessio Gullino, Daniele Ugues

**Affiliations:** 1 Department of Applied Science and Technology (DISAT), Polytechnic University of Turin, Corso Duca degli Abruzzi 24, 10129 Torino, Italy; 2 Consorzio Interuniversitario Nazionale per la Scienza e Tecnologia dei Materiali (INSTM), Via G. Giusti 9, 50121 Firenze, Italy

**Keywords:** dual-phase steel, advanced high-strength steel, cold rolling, bainitic–ferritic steel, Kernel Average Misorientation (KAM)

## Abstract

A new advanced dual-phase (DP) steel characterized by ferrite and bainite presence in equal fractions has been studied within this paper. The anisotropy change of this steel was assessed as a progressively more severe cold rolling process was introduced. Specifically, tensile tests were used to build a strain-hardening curve, which describes the evolution of this DP steel’s mechanical properties as the thinning level increases from 20 to 70% with 10% step increments. As expected, the cold rolling process increases mechanical properties, profoundly altering the material’s microstructure, which was assessed in depth using Electron Backscatter Diffraction (EBSD) analysis coupled with the Kernel Average Misorientation (KAM) maps. At the same time, the process strongly modifies the material planar anisotropy. Microstructural and mechanical assessment and the Kocks–Mecking model applied to this steel evidenced that a 50% strain hardening makes the DP steel isotropic. The material retains or resumes anisotropic behavior for a lower or higher degree of deformation. Furthermore, the paper evaluated the forming limit of this DP steel and introduced geometric limitations to testing the thin steel plates’ mechanical properties.

## 1. Introduction

The demand for safer and lighter vehicles have led to the development of new steels with increased mechanical properties. In particular, manufacturers are looking for materials with enhanced formability, high strength, and good strain hardening indexes. As a result, the automotive field has started introducing advanced high strength steels (AHSS) [1,2,3,4,5,6,7,8,9,10,11,12,13,14] in constructing several cars components. This material category consists of several kinds of steel, among which transformation-induced plasticity (TRIP), twinning-induced plasticity (TWIP), and dual-phase (DP) are the most widely used. According to Kalashami [15], nowadays, the automotive field has at its disposal at least 20 different types of AHSS with which crucial vehicle parts can be built. In particular, 30% of these new materials belong to the DP category, with an ultimate tensile strength ranging from 500 to 1000 MPa. 

Moreover, the oil and pump field has found DP steels extremely useful, particularly those containing bainite as the reinforcing phase. These steels show high buckling resistance and can withstand the bending deformation caused by earthquakes or permafrost melting. In addition, DP steels are typically enriched in Nb when low-temperature environments are involved in achieving high strength and toughness via a tailored precipitation hardening [16]. In addition, these materials possess superior mechanical properties, which can also be increased by modifying the chemical composition or by traditional straightening mechanisms.

According to the literature, DP steels can be reinforced in several ways: some deal with subtitle modification of their chemistry as proposed by Wang [17], others with thermomechanical treatments as suggested by Ghafar [18]. The former methods consist of adding elements such as Nb, V, Ti, or Mo to steels since they provide solution strengthening, precipitation hardening, and reduce the grain coarsening during the manufacturing process [19]. In the latter case, the methods deal with plastic deformation of steels via hot, warm, or cold rolling [15]. Often, the final material strengthening of the material results from the sum of the two. As documented in the literature [20], the DP steel investigated in this paper has an exceptionally high content of Mn, which hinders the grain growth during intercritical annealing, leading to a very fine-grained structure. Moreover, Mn is also added to improve the steel’s hardenability and provides solid solution straightening. Furthermore, this steel is characterized by the presence of Mo, which, in turn, increases the hardenability, suppresses pearlite formation, and gives precipitation hardening [18]. 

Among AHSS, dual-phase steels are being increasingly studied because they simultaneously combine high strength, ductility, and toughness. Traditionally, this condition cannot be satisfied using standard single-phase steels for automotive applications [11]. The mechanical properties of DP steel are strongly influenced by ferrite-to-martensite (or bainite) ratio, grain size, and morphology. According to Akbarpour [20], yield strength and tensile strength of DP steels can be enhanced by increasing the fraction of the reinforcing phase; on the other hand, elongation will be lower. These steels are typically 50% ferrite and 50% reinforcing phase (martensite or bainite). The mechanism at the base of their manufacturing process is a thermomechanical treatment. By heating the rolled steel in the intercritical region, the initial ferritic/perlitic microstructure is altered by austenite formation. The subsequent quenching to room temperature or use of an isothermal transformation allows the transformation of austenite into martensite or bainite [4,5,6,7,8,10,11,12,14,17,20,21,22,23,24,25]. The heat treatment performed on this DP steel has been described in previous work from this research group [26]. Since these steels have two phases, their formability and mechanical properties are higher, but drawability is lower. This condition is strongly related to the low R-value, which characterizes DP steels [14,27]. In addition, the drawability of DP steels is strongly influenced by their atypical microstructure, particularly by the volume fraction and spacing of the bainitic or martensitic particles. Thus, altering the spacing among the reinforcing phases via strong plastic deformation will alter the planar and normal anisotropy of the material, which was also documented in these papers [14,28,29]. 

Ferrite’s contemporary presence with a harder phase increases the tensile-yield strength ratio, allowing a relatively ductile material [30]. Furthermore, DP steels show a higher work hardening rate with respect to single phase steels [14,30]. Despite this, the mechanical properties of DP steels can also be improved or severely modified if subsequent treatments are performed, such as cold rolling. Typically, DP steels need a further strengthening process before being used in automobile components like members, bumper supports, or pillars. Such techniques aim to increase the material strength while trying to limit the ductility loss [15,31]. According to Shen [32], the grain size reduction induced by a rolling process increases material toughness, especially at low temperatures. For example, Kimura [33] obtained high strength and toughness steels, generating an elongated ultrafine-grained microstructure using a warm rolling process in the ferrite region. The refinement of ferrite martensite or bainite grains can improve the strength and ductility of DP steels. Moreover, according to Wang [34], DP steel mechanical properties are strongly altered by the reinforcing phase’s shape, which rolling can change. In particular, isolated grains lead to superior properties with respect to a chain-like network located aside from the ferrite phase. Nevertheless, no one has yet investigated the effect of these modifications on material isotropy levels.

Moreover, although the literature regarding dual-phase steels is extensive, very little information can be found regarding ferritic and bainitic systems. This lack is even more evident when topics like hardening indexes, anisotropy or work-hardening effects are concerned. Therefore, this paper aims to systematically describe the mechanical behavior of a novel DP steel after being mechanically deformed via cold rolling, a technique which is typically used to obtain the semi-products which are commonly found in car frames or bodies. More specifically, in this study, the effect of a progressively increasing amount of thinning applied to a commercial DP steel was studied in depth. The sheet metal studied in this work was subjected to a 20, 30, 40, 50, and 70% height reduction by cold rolling. The cold-rolled sheets were machined, and samples were mechanically tested. The results were used to assess the steel’s microstructure and isotropy in each condition. Furthermore, this work also aims to define a forming limit for this specific material. Finally, the usage of thin dog-bone specimens was investigated to understand whether mechanical properties could be altered by a non-optimal geometric shape of the tensile specimens. In other words, the paper will explain how the dog-bone samples’ width must be corrected to mitigate the effects of their progressive thinning due to the cold rolling process. This result will demonstrate how measurement errors due to geometrical factors during tensile tests are crucial to avoid. 

## 2. Materials and Methods

### 2.1. Materials and Samples Preparation 

**Figure 1:**
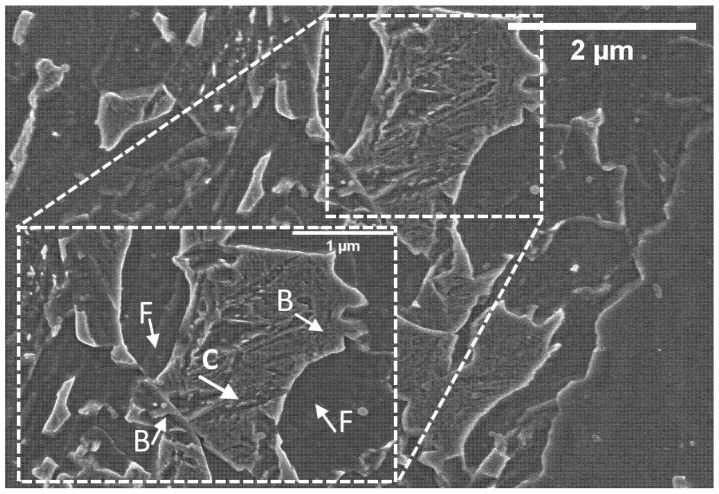
High-resolution micrography showing bainitic islands (indicated as B) surrounded by ferritic matrix (indicated as F). Carbide platelets are indicated with a C.

In this paper, a low-carbon low-alloyed steel, characterized by bainite and ferrite in equal parts, has been carefully characterized. Figure 1, taken at high magnification, shows its microstructure in detail. As described in the literature, ferritic islands are extremely poor in carbon and do not contain precipitated carbides, and are also known as ferrite sheaves. On the other hand, bainitic islands present intense carbide precipitation caused by the strong diffusion of carbon from the ferrite upon its cooling from the A_3_ critical temperature. Furthermore, the insert in the picture also shows the carbide platelets (Fe_3_C, Fe_2–4_C) which characterize the bainitic islands as described by Bhadeshia and Furuhara [35,36]. 

This work presents the DP steel mechanical properties after being cold rolled by a 20, 30, 40, 50, 60, and 70% height reduction. In addition, results allowed the construction of the so-called “strain hardening curves,” i.e., plots indicating the evolution of a specific material property (e.g., E, Ys, UTS, and elongation) as a function of the thinning percentage.

Table 1 shows the average chemical compositions of the steel, expressed in wt% according to the manufacturer datasheets.

The steel sheets were initially provided with a 5.5 mm height after receiving the thermomechanical treatment which stabilized the 1:1 ferritic and bainitic microstructure. The as-received material was then cold rolled to different final thicknesses by a third party company (Itla-Banaiti, Oggiono, Italy). Specifically, their final thicknesses were 5.00, 4.40, 3.85, 3.30, 2.75, 2.20, and 1.65 mm, respectively. First of all, the microstructure of these samples was assessed in the longitudinal and transverse directions with an electronic microscope and EBSD Specimens were prepared according to the standard metallographic procedures to complete this evaluation. Therefore, samples were cut, embedded in resin, and ground with SiC paper down to 2400 grits. Later, samples were polished with diamond pastes up to 1 µm. After each polishing step, samples were rinsed with abundant water and cleaned with ethanol. Microstructures were revealed after etching with nital 4%vol. Finally, a general metallographic observation of samples was performed with Scanning Electronic Microscope SEM MEB Leo 1450 VP (Carl-Zeiss, Oberkochen, Germany). When higher magnification was required, FESEM Zeiss Merlin was equipped with a Gemini column (Carl-Zeiss, Oberkochen, Germany).

### 2.2. Electron Backscatter Diffraction and Kernel Average Matrix Measurements 

Using EBSD, an advanced microstructural assessment was performed on this steel after 20% cold rolling, to identify its metallurgical constituents. The sample was polished with 0.01 μm colloidal silica suspension and it was observed on a plane parallel to the rolling direction. EBSD Maps were performed without etching the sample. A map with a resolution of 256 × 240 pixels, with a step size of 80 nm, was created. The EBSD map was further processed by analyzing the Kernel Average Matrix (KAM), adopting the procedure proposed by Zaefferer et al. [37]. KAM is a measure of local grain misorientation that is usually derived from EBSD data using the following formula:

(1) $${\mathrm{KAM}}_{i,j}=\frac{1}{\left|N\left(i,j\right)\right|}\underset{\left(k,l\in N\left(i,j\right)\right)}{\sum }\omega \left(o_{i,j},o_{k,l}\right)$$

Here, *o_i_*,*_j_* denotes the orientation at pixel position (*i*,*j*) and *N*(*i*,*j*) is the set of neighboring pixels. |*N*(*i*,*j*)| denotes the number of all neighboring pixels taken into account, and *ω*(*o_i_*_,_*_j_*,*o_k_*_,_*_l_*) is the disorientation angle between the orientation *o_ij_* in the center and the neighboring orientation (*o_k_*_,_*_l_*).

Most commonly, the following additional constrains are made:

- consider neighbors up to order n, e.g., n = 1, 2, 3, …;
- consider only neighbors belonging to the same grain;
- consider only neighbors with a misorientation angle smaller than a threshold angle.

According to the work of Zaefferer et al., the KAM is sensitive to phase changes, and has been proved to correctly identify BCC iron phases with progressively higher degree of distortion such as bainite or martensite. Here, the KAM assessment confirmed that the steel contains ferrite and bainite in a 1:1 ratio.

### 2.3. Tensile Tests

Following ISO 6892, all tensile tests were performed with a Zwick Z100 equipped with a 100 kN loading cell (Zwick-Roell Gmbh, Ulma, Germany). Elongation was measured thanks to an extensometer until samples failed. Tensile test properties were performed on specimens machined from the center of the coil with three different metallurgical orientations, i.e., parallel, transversal, and 45° oriented with respect to the rolling direction. Dog-bone samples were machined from all the sheets described in the former paragraph, i.e., with 7 different hardening grades. Lastly, five samples for each combination (thickness + metallurgical orientation) were tested.

During the tensile test, elongation at maximum strength and after the break were measured, respectively. Next, broken specimens were measured to calculate the normal and planar anisotropy according to ASTM E517-2010 standard using Equations (1) and (2), respectively

(2) $$R_{m}=\frac{r_{0}+r_{90}+2r_{45}}{4}$$

(3) $$\Delta R=\frac{r_{0}+r_{90}-2r_{45}}{2}$$

In these Equations, *r* is calculated following [28] using the formula:

(4) r=lnww0lntt0

In this case, *w*_0_, *t*_0_, *w*, and *t*, are the width and the thickness of the tensile samples before and after the tensile test, respectively. The subscript *r* indicates the metallographic orientation of the tested specimen. Even though the specimen size was compliant with ISO 6892, the same standard does not provide any thickness requirement. Thus the impact of such a parameter was addressed by varying the width to thickness ratio of the thinnest tensile samples, as Yuan suggested in his work [38]. 

### 2.4. Nano Hardness Tests

This experiment was performed using a TI950 Nanoindenter (Hysitron, Minneapolis, MN, USA). The most common method to analyze the hardness values from an indentation is the Oliver-Pharr method. The Oliver-Pharr method requires no imaging of the indentation; instead, it is based on contact mechanics [39]. The tests were performed applying a controlled load using a geometrically well-defined probe, producing traditional force versus displacement curves. A diamond Berkovich tip indenter was used, a 3-sided diamond pyramid with a total included angle of 142.3°; another known geometric value is the angle formed between the normal and a face, i.e., 65.35°. The load was increased up to a maximum of 2.5 mN and then decreased at the same rate i.e., 65 μN/s. The indentations were equally spaced using a 2 μm step between each other. A Field Emission Scanning Electron Microscope (FESEM) (Carl-Zeiss, Oberkochen, Germany) was used to assess the 6 × 7 grid performed for a total of 42 indentations. 

## 3. Results

### 3.1. Assessment of Microstructural Properties of DP Steel with Different Strain Hardening Levels

**Figure 2:**
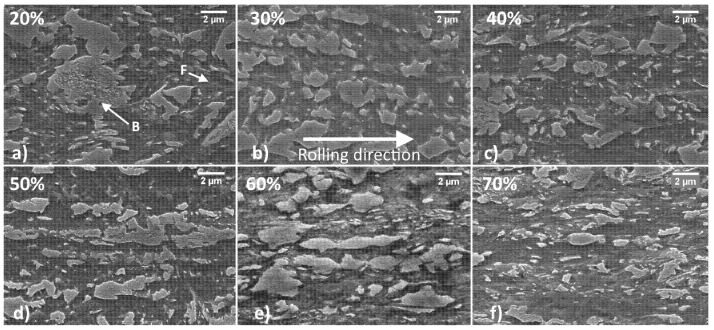
Microstructures of the DP steel as the thinning level is increased, samples with longitudinal orientation observed with SEM. Thinning level is increased as follows 20, 30, 40, 50, 60, 70% represented in (**a**–**f**) panels.

**Figure 3:**
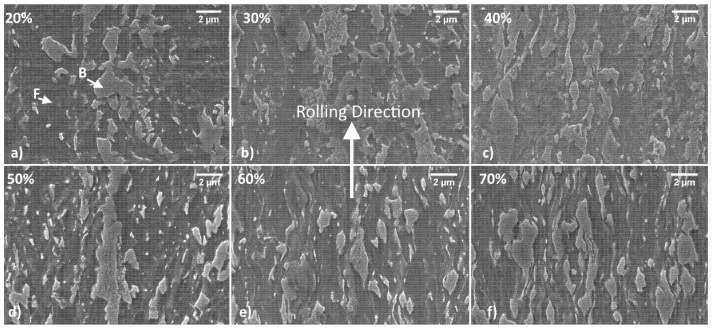
Microstructures of the DP steel as thinning level is increased, samples with transversal orientation observed with SEM. Thinning level is increased as follows 20, 30, 40, 50, 60, 70% represented in (**a**–**f**) panels.

Samples were prepared to observe the microstructure in the longitudinal and transversal direction with respect to the rolling direction with an electronic microscope. The most representative images taken at 10 K x magnifications for each thinning level are shown in Figure 2 and Figure 3. 

Figure 2 shows the samples in the longitudinal orientation. For clarity, letters B and F were used to indicate bainitic and ferritic islands only in the box showing the 20% strain hardening level. Ferritic and bainitic regions always present a similar appearance to Figure 1, although the applied thinning process drastically altered their shape and distribution. The description of these metallurgical constituents has already been introduced in a previous work by Bassini et al. [26] and agrees with Bhadeshia [35], who showed lower bainite surrounded by ferrite. This DP steel shows bainite islands oriented along the rolling direction differently from classically ordered microstructures obtained after thermomechanical treatment. The orientation of the bainitic island increases as the thinning increases. Ferrite is much softer than bainite; thus, it looks strongly deformed along the rolling direction. On the other hand, bainite is harder, and during plastic deformation, this phase can fragment into smaller constituents. 

Figure 3 shows the samples observed along the transversal orientation. The orientation and texture formation are less evident in this sample family. This can be observed comparing the microstructures in the different cold rolling states: the approach of the particles to each other is evident when the thinning range is between 20 and 40% but it is much less evident for higher levels as can be noticed in the second row of the figure.

**Figure 4:**
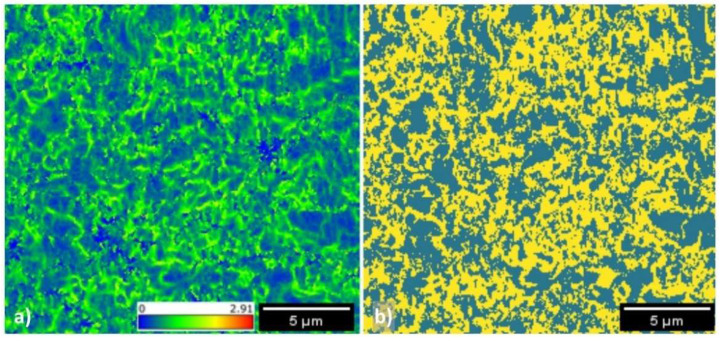
KAM (**a**) as calculated from EBSD detector; and (**b**) after conversion to a binary image using 0.6 as KAM threshold value.

Figure 4a shows the KAM map of the specimen observed in Figure 1. The figure was derived with information obtained from the EBSD detector using Equation (1); a colored map was eventually obtained depending on the degree of misorientation among the neighboring pixels. The computation shows that KAM ranges between 0 and 2.91. The obtained KAM map was further processed and transformed into a binary image (yellow and teal, respectively). This binary image was obtained using a threshold value: more specifically, yellow indicates pixel with a KAM value above 0.6, where a higher misorientation level was observed. Conversely, teal areas had a lower KAM value, indicating a lower misorientation level. Zaefferer et al. effectively used this threshold value to separate ferrite from bainite and martensite. The final result is visible in Figure 4b. All the regions with misorientation values below 0.6 indicate a homogeneous metallurgical constituent without distortion. In other words, the teal regions can be indicated as ferritic domains; conversely, yellow regions have a higher degree of distortion and pixels are characterized by a higher degree of misorientation. According to Zaefferer’s model, the KAM value obtained with this material is compatible with bainite since martensite generally has a KAM higher than 3. 

**Figure 5:**
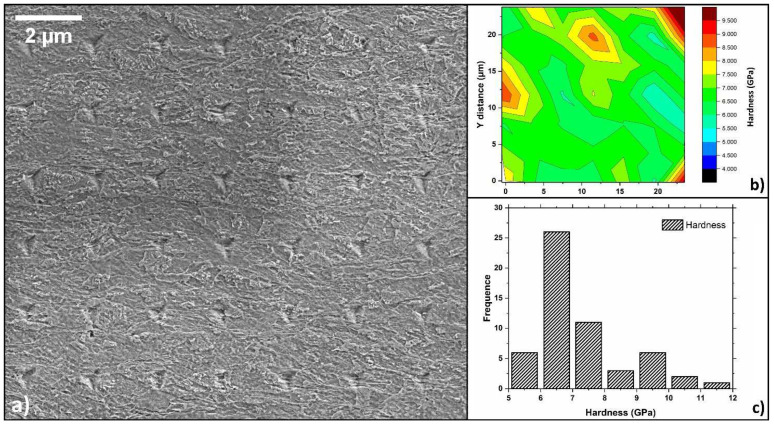
(**a**) Nano-hardness grid and (**b**) the resulting contour map. (**c**) Summary of results from (**a**,**b**) in a histogram showing a bimodal distribution.

The difference mentioned above, between the island families, was further proved thanks to a nano-hardness test. The trial was performed on a sample with a 20% work hardening level to pick the contribution from ferrite and bainite selectively. This choice was important because the sensibility of the test becomes less efficient as the thickness reduction increases. Figure 5 shows where the indentation grid was performed, and the resulting hardness map: different hardness values were obtained depending on the indented particles. The hardest regions correspond to places where the Berkovich indenter interacted with a bainitic island. The 42 hardness values were then assessed via a normality test, confirming that the results do not belong to a Gaussian distribution. When hardness values are plotted in a histogram, as in Figure 5c, two peaks appear, indicating a bi-modal data distribution. The lower hardness peak can be correlated to ferrite islands (6.5 GPa), while the other (9.5 GPa) to bainite; these results are in good agreement with those found for fully bainitic steel by Akram et al. [40]. 

**Figure 6:**
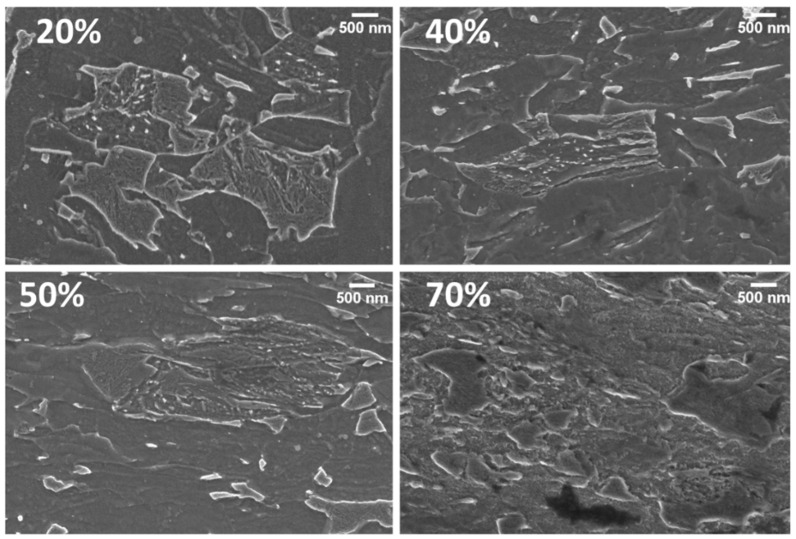
Bainite fragmentation: carbides appear more homogenously distributed as the strain hardening level is increased.

Reorganization of bainitic islands can be better observed at higher magnification, as represented in Figure 6. This picture, indeed, shows samples cold worked at 20, 40, 50, and 70%. It can be observed that, beyond a 50% work hardening level, the islands containing carbides are severely reduced in size.

Furthermore, at this magnification, it is possible to appreciate the ferritic grains’ elongation and the partial fragmentation of bainitic islands as the work hardening increases. When thinning level ranges between 40 and 50%, the metallurgical constituents undergo a progressive homogenization within the overall microstructure. Applying a higher deformation level brings the bainitic islands closer, with a subtle layer of ferrite in between. This condition brings several islands together, leading the bainitic island to behave as a single entity with a similar size to that observed for lower thinning levels. As a result of this microstructural reorganization, mechanical properties, especially those related to anisotropy, were further modified. 

**Figure 7:**
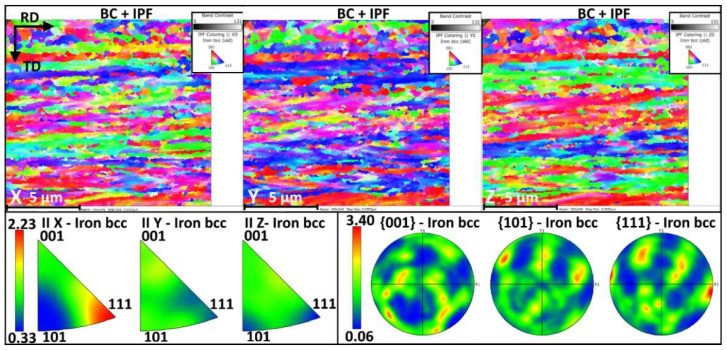
DP steel after 50% cold rolling observed with EBSD. Grains were observed in the x, y and z directions. The texture due to cold rolling is clearly visible. Pole figures and IPF are also shown in the bottom part of the figure.

When a 50% thinning level is achieved, the bainitic islands are dispersed into the ferritic matrix, as shown by the EBSD maps in Figure 7. The maps show bainitic and ferritic grains with a relatively similar size having the rolling direction parallel to the X-axis of the reference system of the maps. According to EBSD maps, many grains share the same crystallographic orientation, contributing to forming ribbons. The inverse pole figures (IPF) show different situations depending on the specific map orientation. Along X, many grains have their 001 and 111 directions parallel to the rolling direction. The Y map again shows many bands with the same orientation, more specifically, many grains show the 111 direction parallel to Y. Finally, the Z map again shows a banded microstructure where grains have the 101, 001 and 111 parallel to the Z direction. This condition is well summarized in the dedicated IPF triangles. Looking at these plots, it is possible to note a preferential alignment in the 111 direction of the bcc cell parallel to the x direction with a mud level close to 2.23.

### 3.2. Analysis of Tensile Properties 

**Figure 8:**
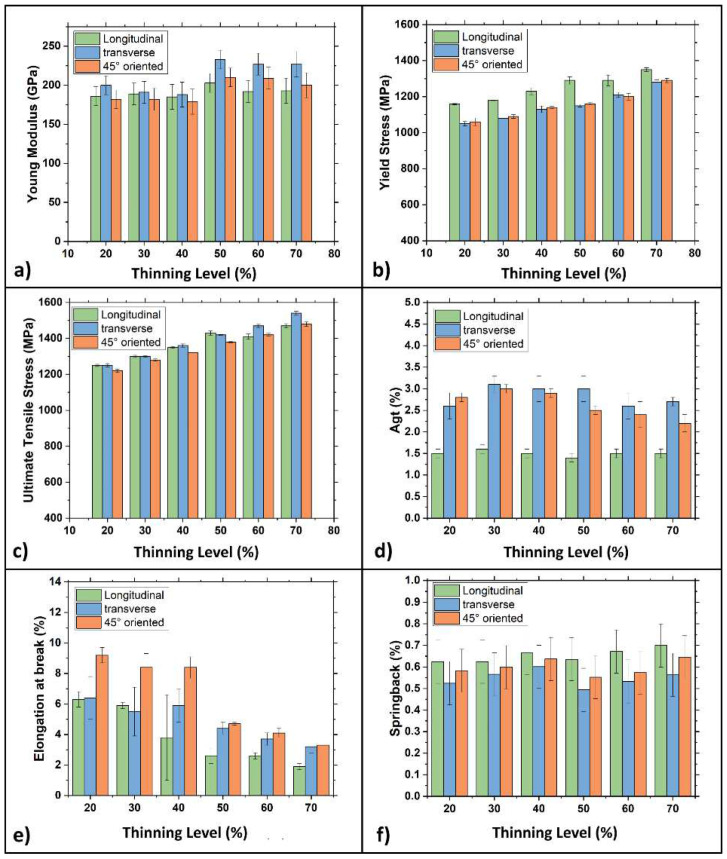
Bar plots of the mechanical properties of the DP steel in all three metallurgical orientations as a function of the thinning levels. (**a**) Young modulus; (**b**) yield stress; (**c**) ultimate tensile stress; (**d**) elongation before sample striction; (**e**) elongation at break; (**f**) springback effect.

Figure 8 graphically shows the mechanical properties measured on samples with three different metallurgical orientations, i.e., parallel, transverse, and 45° tilted, regarding the rolling direction per work hardening level. 

**Figure 9:**
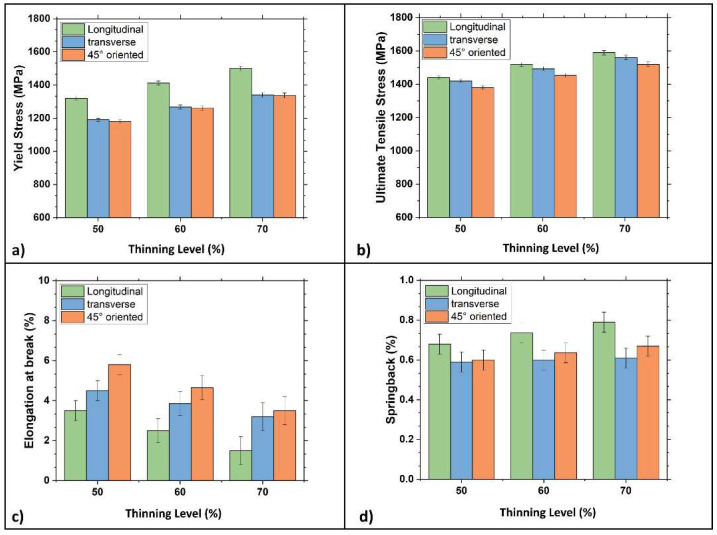
Mechanical properties as a function of metallurgical orientation after thinning the DP steel by 50, 60 and 70% with w/t = 4.5. (**a**) yield stress, (**b**) ultimate tensile stress, (**c**) elongation ant break and (**d**) springback effect.

Thanks to the data obtained from the tensile tests, it is possible to describe the effects of the rolling process on the material. Yield and ultimate stress increase as the work hardening level increases; this condition is evidenced in all the metallurgical orientations. Figure 8a shows how the Young modulus measured in the cross direction is always higher than in the other two metallurgical orientations. More specifically, differences became more evident as the 50% work hardening level was exceeded. For these samples, a substantial increase of the Young modulus is also observed in the 45° tilted direction. It is worth noting that these values are even higher than those surveyed in the longitudinal direction. Figure 8b,c show how, up to 40% of thickness reduction, the yield stress changes a lot depending on the samples’ metallurgical orientation. This difference becomes smaller as the work hardening increases, almost disappearing at the highest thickness reduction, i.e., 70%. Ultimate tensile strength in all three metallurgical directions is similar up to 50% thickness reduction. In contrast, cold-rolled samples with 60 and 70% thickness reduction show a more marked difference with a higher resistance level observed along the transversal direction. Figure 8e shows elongation at break: in the 45° tilted direction, a break is consistently higher than in the other two directions and progressively decreases as the work hardening increases. In particular, beyond a 50% thickness reduction, elongation becomes lower than 4%. Based on these results, the springback forces have been calculated and plotted in Figure 8f. Again, the springback effect changes as a function of the metallurgical orientation: it is more evident in the longitudinal direction while weakest in the transversal one. It is noteworthy that the width/thickness (w/t) ratio was higher in samples with a work hardening level of 50, 60, and 70% than in specimens with lower work hardening levels. For example, for samples with a 20, 30, and 40% strain hardening level, the w/t ratio ranged from 2 to 4. On the other hand, the ratio was higher and ranged from 7 to 11 in samples with a higher work hardening level. The abrupt variation of mechanical properties for samples with a very high w/t ratio might confirm that mechanical properties have been altered by geometrical factors profoundly. According to the literature [38,41], using a w/t ratio below 20 guarantees that the tensile test is performed in plane strain conditions. Nevertheless, using a ratio higher than 8 may lead to a localized necking of samples, resulting in a wrong interpretation of experimental data. Keeping this in mind, specimens with a 50 and 70% thickness reduction were machined to obtain tensile samples with a w/t ratio ranging between 4 and 7.5, aiming to separate the cold rolling effects from those related to the specimen’s geometry. Figure 9 shows the samples’ mechanical properties with 50, 60, and 70% thickness reduction after modifying their w/t ratio. This modification didn’t change the samples’ mechanical properties regardless of the metallurgical orientation, except for the yield and the ultimate stress in the longitudinal direction, which increased with a decreasing w/t ratio. This fact is particularly evident for samples with a 70% thickness reduction. For samples with different metallurgical orientations, differences are much more limited. Finally, elongation at break was unaltered by the change of the sample geometry. 

### 3.3. Strain-Hardening Curves

**Figure 10:**
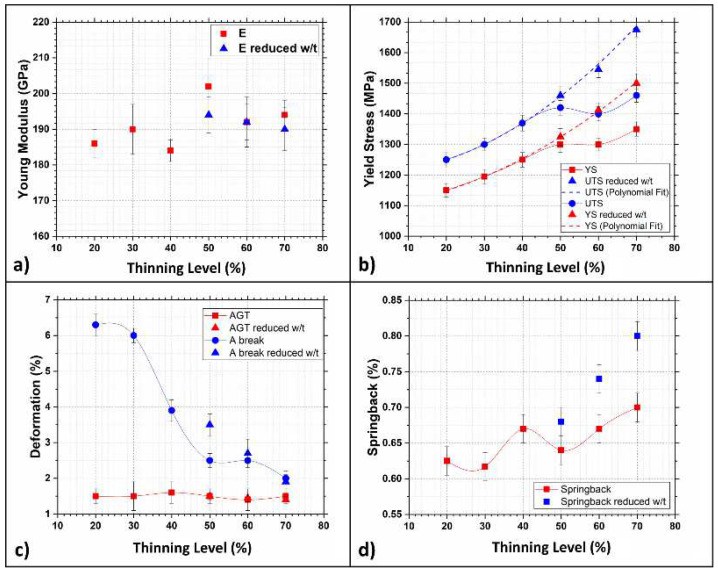
Evolution of mechanical properties and springback for longitudinal samples as a function of strain hardening level (orange circles and squares indicate samples with reduced w/t ratio). (**a**) Young modulus, (**b**) yield stress, (**c**) deformation and (**d**) springback effect.

Only longitudinal samples, with the metallurgical orientation parallel to the rolling direction, RD, have been used to build the strain-hardening curves, these being the results of most interest for future industrial applications. These plots correlate the most relevant mechanical properties with the thinning level of the samples. Figure 10 shows these plots, which also consider the effect of a variation of the w/t ratio. The Young modulus, Figure 10a, shows a fluctuation around 50% thinning level, which is strongly reduced by varying the w/t ratio of samples. According to the strain hardening curves in Figure 10b, YS and UTS regularly increase until the 50% thinning level is reached, independent of the w/t ratio. Beyond this level, samples with w/t higher than 8 present a singularity, forming a local maximum between 40 and 60%. For higher reduction levels, values resume an increasing trend, with a slope similar to that previously observed. More specifically, the samples with a 50% thinning level show comparable YS and UTS values independently of the w/t ratio used, indicating that geometrical parameters still play a limited role at this stage. On the other hand, tensile properties are considerably higher in samples with a 60 and 70% strain hardening level if the w/t ratio is reduced. It is noteworthy that after modifying the sample shape, a single second-order polynomial expression can interpolate all the experimental points obtaining an R^2^ value as high as 0.998. This fact may suggest that the w/t ratio strongly alters the tensile properties of very thin samples because they pass from a plane strain to a plane stress state [42]. Thus, results will show the fluctuations mentioned above if the w/t ratio of the samples is neglected. Nevertheless, this instability is strongly reduced if the w/t ratio is modified accordingly. 

Figure 10c shows elongation at break, which passes from 6 to 3% when the thinning level ranges from 40 to 60%; at this point, a plateau level is reached. Here the w/t ratio only slightly alters the overall results. Conversely, the thinning level does not affect Agt, i.e., the deformation at maximum tensile stress. Moreover, Figure 10d shows a singularity in the springback curve when the strain hardening ranges between 40 and 60%. Finally, samples with a reduced w/t show higher springback values. According to the experimental results, the unexpected values observed in the strain hardening curves should primarily be attributed to the geometry of the samples. In particular, high w/t ratios lead to the formation of localized necking during the tensile test. Thus, a notching effect is applied to the samples, which, in turn, bear the load less effectively. Nevertheless, other parameters, such as the elongation at break, are not influenced by the w/t ratio, which monotonously decreases once a strain hardening level of 40% is reached. Finally, the springback effect increases in both sample categories as the strain hardening increases. More precisely, the increasing trend is more evident if a reduction of the w/t ratio is applied.

**Figure 11:**
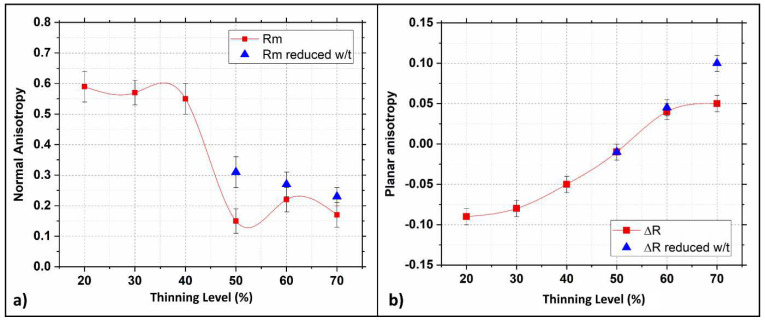
Anisotropy measured using Equations (1), (2) and (3): (**a**) Normal R and (**b**) planar ΔR.

After tensile tests, normal and planar anisotropy levels were measured by collecting the samples’ thickness and width. Calculated levels are plotted in Figure 11 as a function of the strain hardening levels.

Figure 11a shows that the normal anisotropy-value R strongly decreases beyond 40% strain hardening. Moreover, samples with reduced w/t show a smoother variation of R for thinning levels ranging between 40 and 70%. This evidence proves that the samples’ geometry also plays an important role in this case. On the other hand, the average planar anisotropy (ΔR) was practically unaffected by modifying the w/t ratio, as shown in Figure 11b. Despite this, it is essential to stress how the thinning level affects planar anisotropy, which becomes zero and then inverts its sign once 50% thinning is reached. Furthermore, for a correct interpretation of plots in Figure 11, it is essential to remember that R gives a formability index; high R values indicate a high resistance to thinning of metal sheets, while lower ones indicate enhanced formability. Thus, this dual-phase steel can be deformed more easily by regulating the microstructure, making it more homogeneous. On the other hand, ΔR indicates the tendency of earing formation during sheet metal forming. When ΔR is 0, the metal sheet is immune to earing; if negative or positive, the earing forms with a different position relative to the rolling direction. According to the results, ΔR continuously increases, and the switch from negative to positive is observed for a strain hardening level of 50%. This fact suggests that the cold rolling process makes the DP steel microstructure progressively more homogenous. This idea is in good agreement with the microstructure assumed by the DP steel after the cold rolling process. The bainitic islands are smaller and homogeneously dispersed when a thinning level of 50% is reached. This process forms a more isotropic structure, thus leading ΔR to approach 0. By increasing the hardening level, bainitic islands tend to form larger blocks leading to a progressive increase of planar anisotropy. 

### 3.4. Analysis of Fracture Surfaces 

**Figure 12:**
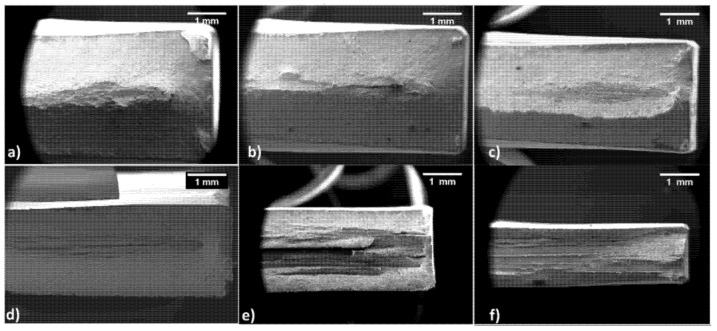
Fractured surface of longitudinal samples with increasing work hardening levels of (**a**) 20, (**b**) 30, (**c**) 40, (**d**) 50, (**e**) 60 and (**f**) 70%.

Figure 12 shows fracture surfaces for longitudinal samples. The innermost part of all samples is profoundly different from the rest of the fractured area. The central part is flat with traces of delamination; externally, a slant fracture is visible, and dimples can be observed. In particular, starting from the 20% thinning level in Figure 12a, a ladder-like fracture is visible with evident delamination phenomena. As the strain hardening level increases, the flat fracture mode with delamination becomes more apparent. In contrast, the surface occupied by the slant fracture is progressively reduced and almost suppressed in the sample tested with a 70% thinning level. Moreover, with the increase of hardening, the delaminated layers’ height and grooves became progressively thicker and more profound, respectively. 

**Figure 13:**
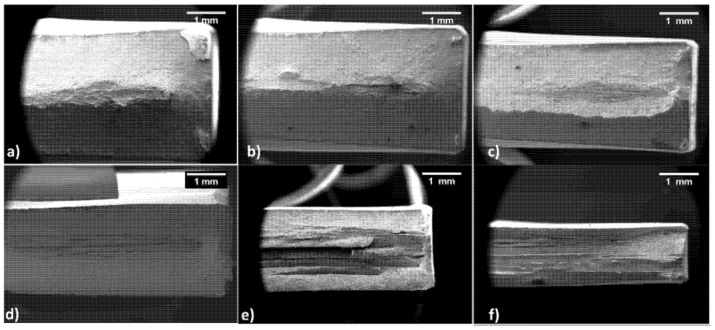
Fractured surface of transversal samples with increasing work hardening levels of: (**a**) 20, (**b**) 30, (**c**) 40, (**d**) 50, (**e**) 60 and (**f**) 70%.

Figure 13 shows the evolution of the fractured surfaces of transverse samples. As previously observed at lower strain hardening levels, the flat and slant fracture are distinguishable. On the other hand, the amount of flat and delaminated fractures increases rapidly, as shown in Figure 13b. Conversely, as expected, Figure 13c shows a reduction of the flat and delaminated fracture surface. Despite this, the fracture is entirely contained in the same plane. If the strain hardening level increases, as in Figure 13d,e, the fracture is much more complex: half the sample presents the usual fracture mode with delamination at the center and slant fracture externally. In contrast, the other half displays a strongly inclined fracture on a plane tilted by ca. 45°. Finally, Figure 13f shows a brittle fracture without delamination, strongly opposing longitudinal samples.

The different nature of bainitic and ferritic islands is at the base of the reinforcing of dual-phase steel. Nevertheless, the harder bainitic island could act as a stress raiser point after an extreme alteration of the material, as in the 70% thickness reduction case. During the uniaxial tensile test, the junction point between ferrite and bainite may cause a preferential crack initiation point. Once the crack is formed, it is free to propagate along the textured material, running at the ferrite or bainite grain boundaries. This phenomenon can happen simultaneously in different portions of the tensile sample leading to a banded fracture surface. The fact that delamination is immediately visible in the centre of the fracture surface may be due to an interaction between the stress raiser points (at the interface between ferrite and bainite) and nucleation of pores which are form the basic mechanism of tensile specimen failure. Similar conclusions were also found in the work by Jiang et al. [43] and in the one from Varanasi et al. [44], albeit for a DP steel with a different microstructure containing martensite. 

## 4. Discussion

### 4.1. Bainitic Island Size Reduction and Anisotropy Modifications

**Figure 14:**
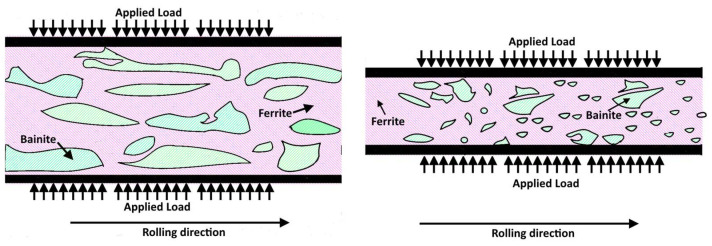
Schematic showing the change in shape and organization of bainitic islands in ferrite matrix passing from 20 to 50% strain hardening level.

By increasing the work hardening level, the steel undergoes severe microstructural alterations. Ferrite can be strongly deformed, flowing along the rolling direction. Bainite, on the other hand, is rigid and can only be slightly distorted. Beyond a certain deformation level, bainite is progressively reduced in size; its shape passes from elongated to rounded. The consequence of this fragmentation is that the material becomes progressively more uniform since the two phases (ferrite and bainite) combine homogeneously. This microstructural homogenization reaches a maximum approaching 50% of cold working. Beyond this point, bainite islands tend to form larger blocks resulting in a microstructure similar to the original one. This fact agrees with the observations at high magnification with the FESEM. The combination of these events explains the evolution of planar anisotropy as a function of the thinning level. A 50% section reduction leads to a homogenous dispersion of smaller bainitic islands surrounded by soft ferrite. This way, the material reaches a planar anisotropy level close to zero. Thus, it can be drawn without incurring the earing phenomenon. This condition is schematized in Figure 14, showing how the microstructure changes as the strain hardening increases from 20 to 50%.

Bainite becomes the homogeneously dispersed reinforcing particle system of the alloy. According to the other mechanical properties collected during this work, this thinning level also corresponds to the material’s forming limit. In fact, despite mechanical properties—such as the yield or the ultimate tensile stress—increasing as the strain hardening increases, elongation at break becomes extremely low. Furthermore, the morphological investigation proved that the steel becomes progressively more brittle, as demonstrated by the increasing number of delaminated plates.

### 4.2. Use of Kocks–Mecking Model to Evaluate the Strain Hardening Rate 

**Figure 15:**
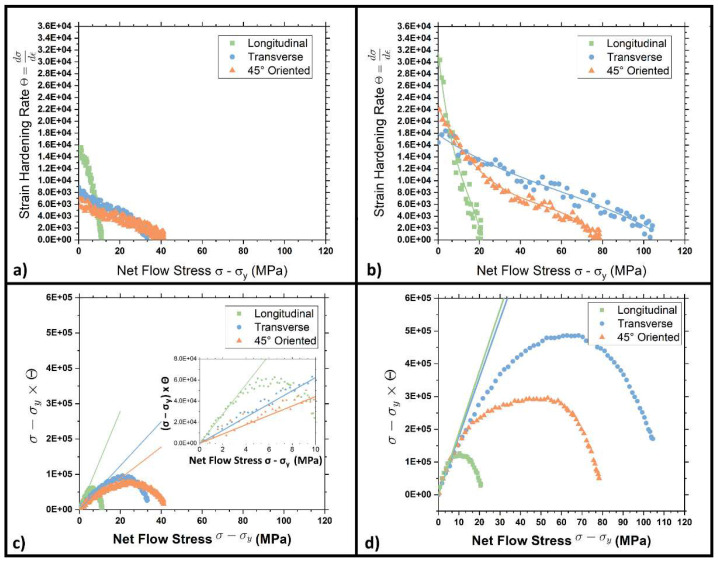
Strain hardening rate and net flow stress plots for samples with (**a**) 20 and (**b**) 50% strain hardening level; and (σ − Y)Θ versus net flow stress (σ − Y) plots for DP steel with (**c**) 20 and (**d**) 50% strain hardening level.

**Figure 16:**
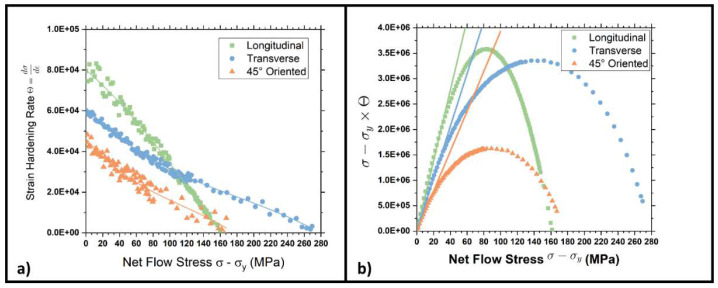
(**a**) Strain hardening rate and net flow stress plots and (**b**) (σ − Y)Θ versus net flow stress (σ − Y) for samples with 70% strain hardening level.

Mechanical properties of the cold-rolled steel were also assessed via the Kocks–Mecking model in three different conditions. This model was used to measure the strain hardening rate as the cold rolling level was increased. As will be explained below, this approach was fundamental to evaluating the isotropy level of the steel as a function of the applied cold rolling level. These plots were obtained for the steel with a section reduction of 20, 50, and 70% in Figure 15a,b and Figure 16b, respectively. They show a plot of Θ, the strain hardening rate calculated according to Equation (4) as a function of the net flow stress (σ − σ_y_):

(5) $$\theta =\frac{d\sigma }{d\varepsilon }$$

where *σ* and *ε* are the true stress and true strain, respectively, and σ_y_ is the yield stress. 

According to the literature [45,46], if a linear strain hardening behavior is present, the Kocks–Mecking plot should show an initial plateau known as II stage strain hardening. The studied steel, on the contrary, as shown in Figure 15 and Figure 16, shows a decreasing strain hardening rate, thus the so-called III stage. Longitudinal samples show a linearly decreasing trend without any significant slope changes. On the other hand, transversal and 45° oriented tensile specimens indicate changes in the curves’ slope for very high net flow stress. This change in slope indicates the onset of the so-called IV stage of strain-hardening. Moreover, the initial strain hardening rate strongly increases as the section reduction percentage is increased.

Previously obtained data can be further treated by introducing the $\Theta \cdot \left(\sigma -Y\right)$ vs $\left(\sigma -Y\right)$ plots. According to the literature [27,29,38,39], this kind of chart allows establishing a quantitative index of the anisotropy grade of the material. The initial part of the plot of Figure 15c,d and Figure 16b can be interpolated by a straight line, passing through the origin, whose slope Θ_h_ is strongly dependent on the interaction between the matrix and the reinforcing particles [46]. Isotropic materials are characterized by three curves with a similar slope in the plot’s initial part, independent to the test direction. On the other hand, if a material shows anisotropy, the curves’ slope changes accordingly with the test direction. This feature is caused by a different interaction between the dislocation and the reinforcing particles (the bainitic islands in this specific case). The figures show a strong anisotropy in the DP steel when low or very high strain hardening levels are applied (orange, blue, and green lines). The measured Θ_h_ is always higher for longitudinal samples (green), while the lowest values are provided by 45° oriented ones (orange). This evidence was also highlighted with an inset showing the plots closer to the origin. Conversely, by applying a 50% strain hardening level, Θ_h_ assumes values extremely close to each other. According to the explanation given above, the reinforcing system is now acting homogeneously, which is in substantial agreement with the micrographs and the values of planar anisotropy in Figure 11b.

Finally, The downward curvature of the (σ − σ_y_)Θ curves at high stresses indicates the onset of the dynamic recovery regime, which corresponds to the declining slope in stress-strain curves [29,46]. 

Figure 16 shows how Θ_h_ resumes a diverging trend as the ferritic and bainitic islands re-organize in larger blocks. In this condition, the plots show three different tangents depending on the metallurgical orientation of the samples, as previously seen in Figure 15c. This fact further reinforces the hypothesis that the material gradually loses homogeneity as the strain hardening level increases.

## 5. Conclusions

In this paper, the study of the evolution of mechanical properties of a dual-phase steel due to a progressive cold rolling process has been developed. This DP steel is produced using a particular thermomechanical process, i.e., a hot rolling process followed by a specific heat treatment, which gives the steel a dual-phase structure. Ferrite (as ductile phase) and bainite (as reinforcing phase) are simultaneously formed. The subsequent cold rolling process modifies the two phases’ distribution with the deformation of ferrite and a progressive crush of bainitic islands. The most important outcomes can be summarized as follows:

- Increasing the strain hardening level enhances material properties, i.e., UTS and yield stress increase, while ductility is lowered.
- The strain hardening curves for the DP steel were obtained, and demonstrate the DP mechanical properties to be affected by the w/t ratio of the tested dog bone samples. This was particularly evident for yield and ultimate tensile stress, while it was less relevant for elongation at break.
- Fracture surfaces are characterized by delamination of the samples’ innermost part, which is even more evident as the strain hardening level increases. The trend is particularly apparent for longitudinal samples, while transversal ones show a less noticeable trend.
- The 50% thickness reduction is considered the forming limit of the material on the base of fractured surfaces analysis and ductility reduction.
- The cold rolling process causes a progressive microstructural reorganization, reducing the material inhomogeneity, as demonstrated by the levels of planar anisotropy, which becomes zero for a 50% strain hardening level. When a higher level of strain hardening is applied, the microstructure once again shows larger bainitic islands and a net increase in anisotropy. This condition was also evidenced using the Θ(σ − σ_y_) vs. (σ − σ_y_) plots derived from the Kocks–Mecking model. At 50% strain hardening, and independent to the sample test direction, the linear part of the DP steel curves all have practically the same slope indicating that the bainitic island reinforces the material in an isotropic manner.

## Figures and Tables

**Table 1:** Average chemical composition of the steel expressed in wt% according to manufacturer datasheets.

C	Si	Mn	Al	Cr	V	Mo
0.09	0.15	1.45	0.02	1.25	0.12	0.4

## Data Availability

The paper already contains all relevant results; the raw data required to reproduce these findings can be shared on request.
